# (*E*)-2-*tert*-Butyl-6-[(naphthalen-1-yl)imino­meth­yl]phenol

**DOI:** 10.1107/S1600536812002395

**Published:** 2012-01-25

**Authors:** Roghayieh Jamjah, Mehdi Nekoomanesh, Roya Zahedi, Gholamhossein Zohuri, Faramarz Afshar Taromi, Behrouz Notash

**Affiliations:** a Iran Polymer and Petrochemical Institute (IPPI), PO Box 14965/115, Tehran, Iran; b Chemistry Group, Amirkabir University of Technology, PO Box 15875-4413, Tehran, Iran; c Department of Chemistry, Faculty of Science, Ferdowsi University of Mashhad, PO Box 1436, Mashhad, Iran; d Department of Polymer Engineering, Amirkabir University, PO Box 15875-4413, Tehran, Iran; e Department of Chemistry, Shahid Beheshti University, G. C., Evin, Tehran 1983963113, Iran

## Abstract

The asymmetric unit of the title Schiff base compound, C_21_H_21_NO, contains two crystallographicaly independent mol­ecules. The dihedral angles between the naphthalene mean plane and the benzene ring are 29.28 (8) and 26.92.(8)° in the two mol­ecules. An intra­molecular O—H⋯N hydrogen bond and weak intra­molecular C—H⋯O hydrogen bonds stabilize the structure of each independent mol­ecule.

## Related literature

For general background to the synthesis and catalytic activity of FI catalysts, see: Matsui & Fujita (2001 ▶); Matsui *et al.* (1999 ▶, 2001 ▶). For related structures, see: Hiller *et al.* (1993 ▶); Darensbourg *et al.* (2005 ▶); Jamjah *et al.* (2011 ▶).
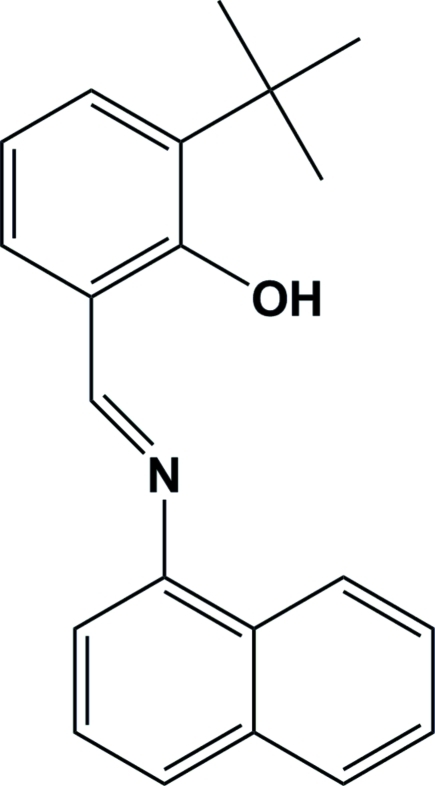


## Experimental

### 

#### Crystal data

C_21_H_21_NO
*M* *_r_* = 303.39
Orthorhombic, 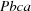

*a* = 9.4770 (19) Å
*b* = 20.109 (4) Å
*c* = 34.595 (7) Å
*V* = 6593 (2) Å^3^
*Z* = 16
Mo *K*α radiation
μ = 0.07 mm^−1^
*T* = 120 K
0.5 × 0.4 × 0.3 mm

#### Data collection

Stoe IPDS II diffractometer
28480 measured reflections
8840 independent reflections
5781 reflections with *I* > 2σ(*I*)
*R*_int_ = 0.071

#### Refinement

*R*[*F* ^2^ > 2σ(*F* ^2^)] = 0.067
*wR*(*F* ^2^) = 0.155
*S* = 1.05
8840 reflections
424 parameters
H-atom parameters constrained
Δρ_max_ = 0.24 e Å^−3^
Δρ_min_ = −0.23 e Å^−3^

### 

Data collection: *X-AREA* (Stoe & Cie, 2005 ▶); cell refinement: *X-AREA*; data reduction: *X-RED32* (Stoe & Cie, 2005 ▶); program(s) used to solve structure: *SHELXS97* (Sheldrick, 2008 ▶); program(s) used to refine structure: *SHELXL97* (Sheldrick, 2008 ▶); molecular graphics: *ORTEP-3 for Windows* (Farrugia, 1997 ▶); software used to prepare material for publication: *WinGX* (Farrugia, 1999 ▶).

## Supplementary Material

Crystal structure: contains datablock(s) I, global. DOI: 10.1107/S1600536812002395/bt5787sup1.cif

Structure factors: contains datablock(s) I. DOI: 10.1107/S1600536812002395/bt5787Isup2.hkl

Supplementary material file. DOI: 10.1107/S1600536812002395/bt5787Isup3.cml

Additional supplementary materials:  crystallographic information; 3D view; checkCIF report

## Figures and Tables

**Table 1:** Hydrogen-bond geometry (Å, °)

*D*—H⋯*A*	*D*—H	H⋯*A*	*D*⋯*A*	*D*—H⋯*A*
O1—H1*A*⋯N1	0.82	1.81	2.552 (2)	150
O2—H2*A*⋯N2	0.82	1.89	2.631 (2)	150
C20—H20*B*⋯O1	0.96	2.38	2.998 (3)	121
C21—H21*B*⋯O1	0.96	2.35	2.987 (3)	124
C41—H41*C*⋯O2	0.96	2.33	2.979 (3)	124
C42—H42*B*⋯O2	0.96	2.41	3.056 (3)	124

**Fig. 1.**
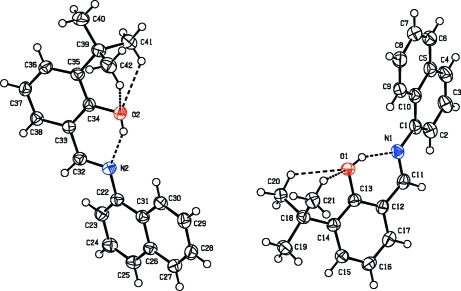
The molecular structure of the title compound with displacement ellipsoids drawn at 50% probability level. The intramolecular O—H···N and C—H···O hydrogen bonds are shown as dashed lines.

## supplementary crystallographic information

### Comment

Since the discovery of post metallocene catalysts systems such as salicylaldimine (phenoxy-imine, FI catalysts) in late 1990's by Fujita group, there have been many researches concerning these catalysts (Matsui & Fujita, 2001; Matsui *et al.*, 2001). In general, they have found that some phenoxy-imine-based complexes of the group IV metals have very high activity, versatility and polymerization capabilities in olefin polymerization (Matsui *et al.*, 1999).

In FI catalysts, the ligand systems can be produced by using condensation reaction of salicylaldehydes and primary amines, under standard conditions. These reactions produce Schiff bases with high selectivity, in high yield. Amines and some salicylaldehyde derivatives are commercially available. The presence of salicylaldimine unit allows for easy variation of the system on both the phenol and amine functionalities, for instance the variations of the mentioned compounds lead to profound effects on polymers.

X-ray crystallographic analysis reveals that there are two molecules in the asymmetric unit of the title Schiff base compound (Fig. 1). In both molecules, the C═N bond distances are 1.289 (3) and 1.290 (3) Å, respectively. The angle between naphthalene mean plane and phenyl rings are equal to 29.28 (8) and 26.92.(8)°, respectively. Bond distances and angles are in the normal ranges reported for Schiff base compounds (Hiller *et al.*, 1993; Darensbourg *et al.*, 2005; Jamjah *et al.*, 2011). There are intramolecular O—H···N hydrogen bonding between hydroxyl and imine groups. There are also weak intramolecular C—H···O hydrogen bonding which play important role in the stabilization of the crystal structure of the title compound (Fig. 1 & Table 1).

### Experimental

Synthesis of 3-*tert*-butyl-2-hydroxybenzaldehyde

2,6-dimethylpyridine (50.0 mmol, 5.4 g) and SnCl_4_ (10.0 mmol, 2.6 g) were added into 2-*tert*-butylphenol (40.0 mmol, 6 g) in toluene (125 ml) into a 250 ml round-bottom flask which had been already dried and purged with nitrogen and equipped with a stirrer bar. White fumes appeared immediately during the addition and stirring was continued at room temperature for 30 min. The mixture turned yellow, and then dry paraformaldehyde (160.0 mmol, 4.8 g) was added into the reaction solution and stirred at 100°C for 12 hrs. After cooling to room temperature, the yellow reaction mixture was poured into a mixed solution of water (3×100) and diethyl ether (3×50). The yellow precipitate was removed *via* filtration. The filtrate was extracted with diethyl ether (2×100 ml) and the organic layer was washed further with saturated NaCl_(aq)_ (brine) (50 ml). The solution was then dried over anhydrous sodium sulfate. After that, it was concentrated under reduced pressure (in a rotary evaporator) and the residue was purified *via* column chromatography on silica gel, using a mixed solvent of ethylacetate/hexane (*v*/*v*, 5/100) as the mobile phase. The product was isolated as pale yellow oil and characterized by ^1^H-NMR, ^13^C-NMR and FTIR as follows.

^1^H-NMR (400 MHz, in CDCl_3_): δ (p.p.m.) 1.43 (s, 9H C(CH_3_)_3_), 6.95 (s,1*H*, Ar—H), 7.39 (s,1*H*, Ar—H), 7.53 (s,1*H*, Ar—H), 9.85 (s, 1H, CHO), 11.82 (s, 1H, OH).

^13^C-NMR (400 MHz, in CDCl_3_): δ (p.p.m.) 30.04 (*t*-Bu—CH_3_), 35.66 (*t*-Bu—C), 120.06 (Aryl-C-5), 121.83 (Aryl-C-1), 132.81 (Aryl-C-4), 134.24 (Aryl-C-6), 162.38 (Aryl-C-2), 197.28 (CHO).

FTIR (KBr): 2958.49 (OH), 1650.66 (CHO) cm^-1^. CHN (Calcd. (Anal): C, 74.05 (74.15); H, 7.87(7.80), (yield: 85%).

Synthesis of (*E*)-2-tert-butyl-6-((naphthalen-1-ylimino)methyl)phenol:

Into a 100 ml round-bottom flask which had been dried and purged with nitrogen, ethanol (30 ml), α-naphthylamine (9.99 mmol, 1.34 g), 3- *tert* -butyl-2-hydroxybenzaldehyde (7.86 mmol, 1.40 g) and molecular sieve 3Å (5 g) were added. This mixture was refluxed at 78°C for 8 hrs and then stirred at ambient temperature for another 12 hrs. The reaction solution was concentrated under reduced pressure (in a rotary evaporator). The residue was purified *via* column chromatography on silica gel, using a mixed solvent of ethylacetate/hexane (*v*/*v*, 2/98) as the mobile phase to obtain a yellow-orange crystalline solid (yield: 98%). ^1^H-NMR (400 MHz, in CDCl_3_): δ (p.p.m.) 1.54 (s, 9H C(CH_3_)_3_), 6.93–7.79 (m, 11H), 8.30–8.71 (m, 1H), 11.81(s, 1H).

### Refinement

All H atoms were positioned geometrically and refined as riding atoms with O—H = 0.82, *U*_iso_(H) = 1.5*U*_eq_(O) and C—H = 0.93 to 0.96 Å, *U*_iso_(H) = 1.5*U*_eq_(C) for methyl H atoms and 1.2*U*_eq_(C) for the others.

### Crystal data

**Table:**

C_21_H_21_NO	*F*(000) = 2592
*M**_r_* = 303.39	*D*_x_ = 1.223 Mg m^−^^3^
Orthorhombic, *P**b**c**a*	Mo *K*α radiation, λ = 0.71073 Å
Hall symbol: -P 2ac 2ab	Cell parameters from 8840 reflections
*a* = 9.4770 (19) Å	θ = 2.5–29.2°
*b* = 20.109 (4) Å	µ = 0.07 mm^−^^1^
*c* = 34.595 (7) Å	*T* = 120 K
*V* = 6593 (2) Å^3^	Block, orange
*Z* = 16	0.5 × 0.4 × 0.3 mm

### Data collection

**Table:**

Stoe IPDS II diffractometer	5781 reflections with *I* > 2σ(*I*)
Radiation source: fine-focus sealed tube	*R*_int_ = 0.071
graphite	θ_max_ = 29.2°, θ_min_ = 2.5°
Detector resolution: 0.15 mm pixels mm^-1^	*h* = −10→12
rotation method scans	*k* = −27→23
28480 measured reflections	*l* = −39→47
8840 independent reflections	

### Refinement

**Table:**

Refinement on *F*^2^	Secondary atom site location: difference Fourier map
Least-squares matrix: full	Hydrogen site location: inferred from neighbouring sites
*R*[*F*^2^ > 2σ(*F*^2^)] = 0.067	H-atom parameters constrained
*wR*(*F*^2^) = 0.155	*w* = 1/[σ^2^(*F*_o_^2^) + (0.0561*P*)^2^ + 2.5641*P*] where *P* = (*F*_o_^2^ + 2*F*_c_^2^)/3
*S* = 1.05	(Δ/σ)_max_ = 0.001
8840 reflections	Δρ_max_ = 0.24 e Å^−^^3^
424 parameters	Δρ_min_ = −0.23 e Å^−^^3^
0 restraints	Extinction correction: *SHELXL97* (Sheldrick, 2008), Fc^*^=kFc[1+0.001xFc^2^λ^3^/sin(2θ)]^-1/4^
Primary atom site location: structure-invariant direct methods	Extinction coefficient: 0.0039 (3)

### Special details

**Table:**

Geometry. All e.s.d.'s (except the e.s.d. in the dihedral angle between two l.s. planes) are estimated using the full covariance matrix. The cell e.s.d.'s are taken into account individually in the estimation of e.s.d.'s in distances, angles and torsion angles; correlations between e.s.d.'s in cell parameters are only used when they are defined by crystal symmetry. An approximate (isotropic) treatment of cell e.s.d.'s is used for estimating e.s.d.'s involving l.s. planes.
Refinement. Refinement of *F*^2^ against ALL reflections. The weighted *R*-factor *wR* and goodness of fit *S* are based on *F*^2^, conventional *R*-factors *R* are based on *F*, with *F* set to zero for negative *F*^2^. The threshold expression of *F*^2^ > σ(*F*^2^) is used only for calculating *R*-factors(gt) *etc*. and is not relevant to the choice of reflections for refinement. *R*-factors based on *F*^2^ are statistically about twice as large as those based on *F*, and *R*- factors based on ALL data will be even larger.

### Fractional atomic coordinates and isotropic or equivalent isotropic displacement parameters (Å^2^)

**Table:**

	*x*	*y*	*z*	*U*_iso_*/*U*_eq_	
O1	0.37905 (15)	0.43850 (7)	0.17017 (4)	0.0338 (3)	
H1A	0.4051	0.4727	0.1810	0.051*	
N1	0.39654 (18)	0.52936 (9)	0.22143 (5)	0.0332 (4)	
C14	0.2144 (2)	0.34967 (10)	0.17797 (6)	0.0307 (4)	
C13	0.2750 (2)	0.40972 (10)	0.19115 (6)	0.0300 (4)	
C12	0.2293 (2)	0.44053 (10)	0.22547 (6)	0.0315 (4)	
C9	0.5443 (2)	0.61773 (11)	0.17089 (6)	0.0366 (5)	
H9	0.4867	0.5852	0.1601	0.044*	
C15	0.1068 (2)	0.32295 (11)	0.20058 (6)	0.0334 (4)	
H15	0.0642	0.2837	0.1925	0.040*	
C1	0.4731 (2)	0.58316 (11)	0.23768 (6)	0.0345 (4)	
C2	0.4857 (2)	0.59221 (11)	0.27685 (6)	0.0388 (5)	
H2	0.4367	0.5644	0.2936	0.047*	
C16	0.0602 (2)	0.35241 (11)	0.23474 (6)	0.0367 (5)	
H16	−0.0118	0.3328	0.2490	0.044*	
C17	0.1212 (2)	0.41069 (11)	0.24727 (6)	0.0367 (5)	
H17	0.0910	0.4303	0.2702	0.044*	
C5	0.6381 (2)	0.67555 (11)	0.22723 (7)	0.0364 (5)	
C18	0.2653 (2)	0.31619 (10)	0.14061 (6)	0.0324 (4)	
C11	0.2939 (2)	0.50132 (11)	0.23963 (6)	0.0349 (5)	
H11	0.2604	0.5204	0.2623	0.042*	
C10	0.5499 (2)	0.62500 (10)	0.21151 (6)	0.0336 (4)	
C4	0.6460 (2)	0.68339 (12)	0.26778 (7)	0.0416 (5)	
H4	0.7028	0.7166	0.2781	0.050*	
C8	0.6230 (2)	0.65808 (12)	0.14724 (7)	0.0413 (5)	
H8	0.6184	0.6524	0.1206	0.050*	
C6	0.7170 (2)	0.71633 (12)	0.20179 (7)	0.0410 (5)	
H6	0.7745	0.7495	0.2119	0.049*	
C7	0.7101 (2)	0.70772 (12)	0.16279 (7)	0.0436 (5)	
H7	0.7631	0.7347	0.1465	0.052*	
C3	0.5715 (3)	0.64292 (12)	0.29195 (7)	0.0424 (5)	
H3	0.5776	0.6489	0.3186	0.051*	
O2	1.00404 (15)	0.13532 (7)	0.03168 (5)	0.0366 (3)	
H2A	0.9468	0.1166	0.0457	0.055*	
N2	0.89339 (17)	0.04113 (9)	0.07488 (5)	0.0322 (4)	
C34	1.1074 (2)	0.09186 (10)	0.02161 (6)	0.0297 (4)	
C22	0.7909 (2)	0.01274 (10)	0.10001 (6)	0.0303 (4)	
C31	0.6520 (2)	0.04053 (10)	0.09993 (6)	0.0306 (4)	
C35	1.2155 (2)	0.11257 (10)	−0.00385 (6)	0.0308 (4)	
C33	1.1031 (2)	0.02646 (10)	0.03615 (6)	0.0296 (4)	
C37	1.3157 (2)	0.00065 (11)	0.00143 (6)	0.0324 (4)	
H37	1.3866	−0.0292	−0.0052	0.039*	
C38	1.2083 (2)	−0.01867 (10)	0.02546 (6)	0.0313 (4)	
H38	1.2053	−0.0621	0.0347	0.038*	
C32	0.9909 (2)	0.00304 (11)	0.06118 (6)	0.0314 (4)	
H32	0.9891	−0.0418	0.0677	0.038*	
C29	0.4784 (2)	0.11944 (11)	0.07694 (6)	0.0370 (5)	
H29	0.4551	0.1558	0.0616	0.044*	
C39	1.2191 (2)	0.18265 (11)	−0.02143 (6)	0.0342 (4)	
C23	0.8228 (2)	−0.03859 (11)	0.12478 (6)	0.0349 (4)	
H23	0.9142	−0.0553	0.1254	0.042*	
C26	0.5478 (2)	0.01151 (11)	0.12424 (6)	0.0329 (4)	
C36	1.3179 (2)	0.06532 (11)	−0.01294 (6)	0.0323 (4)	
H36	1.3911	0.0776	−0.0294	0.039*	
C25	0.5844 (2)	−0.04207 (11)	0.14872 (6)	0.0375 (5)	
H25	0.5161	−0.0611	0.1646	0.045*	
C30	0.6139 (2)	0.09558 (10)	0.07665 (6)	0.0333 (4)	
H30	0.6814	0.1157	0.0610	0.040*	
C24	0.7194 (2)	−0.06625 (11)	0.14925 (6)	0.0377 (5)	
H24	0.7429	−0.1010	0.1658	0.045*	
C28	0.3743 (2)	0.08973 (11)	0.10011 (7)	0.0391 (5)	
H28	0.2823	0.1058	0.0996	0.047*	
C27	0.4082 (2)	0.03728 (11)	0.12329 (7)	0.0377 (5)	
H27	0.3389	0.0181	0.1387	0.045*	
C20	0.4227 (2)	0.29752 (11)	0.14468 (6)	0.0372 (5)	
H20A	0.4357	0.2710	0.1675	0.056*	
H20B	0.4782	0.3373	0.1467	0.056*	
H20C	0.4521	0.2727	0.1224	0.056*	
C19	0.1829 (3)	0.25239 (11)	0.13220 (7)	0.0413 (5)	
H19A	0.1950	0.2217	0.1532	0.062*	
H19B	0.2175	0.2327	0.1088	0.062*	
H19C	0.0846	0.2627	0.1293	0.062*	
C41	1.2218 (2)	0.23574 (11)	0.01050 (7)	0.0419 (5)	
H41A	1.2248	0.2791	−0.0010	0.063*	
H41B	1.3038	0.2294	0.0264	0.063*	
H41C	1.1385	0.2318	0.0261	0.063*	
C42	1.0883 (2)	0.19260 (12)	−0.04681 (7)	0.0426 (5)	
H42A	1.0888	0.2368	−0.0573	0.064*	
H42B	1.0048	0.1864	−0.0315	0.064*	
H42C	1.0892	0.1608	−0.0675	0.064*	
C40	1.3494 (3)	0.19301 (13)	−0.04700 (8)	0.0484 (6)	
H40A	1.3469	0.1623	−0.0682	0.073*	
H40B	1.4332	0.1856	−0.0320	0.073*	
H40C	1.3498	0.2377	−0.0568	0.073*	
C21	0.2438 (3)	0.36255 (11)	0.10589 (6)	0.0392 (5)	
H21A	0.2753	0.3407	0.0828	0.059*	
H21B	0.2972	0.4026	0.1097	0.059*	
H21C	0.1455	0.3733	0.1035	0.059*	

### Atomic displacement parameters (Å^2^)

**Table:**

	*U*^11^	*U*^22^	*U*^33^	*U*^12^	*U*^13^	*U*^23^
O1	0.0327 (7)	0.0332 (8)	0.0354 (8)	−0.0053 (6)	0.0035 (6)	−0.0012 (6)
N1	0.0321 (9)	0.0290 (9)	0.0386 (9)	0.0032 (7)	−0.0038 (7)	−0.0008 (7)
C14	0.0268 (9)	0.0322 (11)	0.0331 (10)	0.0016 (8)	−0.0010 (8)	0.0044 (8)
C13	0.0265 (9)	0.0304 (10)	0.0331 (10)	0.0026 (8)	0.0003 (8)	0.0051 (8)
C12	0.0280 (9)	0.0320 (11)	0.0344 (10)	0.0052 (8)	−0.0004 (8)	−0.0003 (8)
C9	0.0374 (11)	0.0305 (11)	0.0418 (12)	0.0031 (9)	−0.0029 (9)	−0.0054 (9)
C15	0.0322 (10)	0.0300 (10)	0.0380 (11)	−0.0003 (8)	0.0009 (8)	0.0014 (9)
C1	0.0333 (10)	0.0282 (10)	0.0420 (11)	0.0056 (8)	−0.0053 (9)	−0.0050 (9)
C2	0.0436 (12)	0.0350 (12)	0.0378 (11)	0.0016 (9)	−0.0043 (9)	−0.0020 (9)
C16	0.0303 (10)	0.0393 (12)	0.0404 (11)	0.0023 (9)	0.0057 (8)	0.0055 (9)
C17	0.0340 (10)	0.0414 (12)	0.0347 (11)	0.0083 (9)	0.0031 (8)	0.0009 (9)
C5	0.0340 (10)	0.0317 (11)	0.0434 (12)	0.0047 (8)	−0.0053 (9)	−0.0046 (9)
C18	0.0360 (10)	0.0293 (10)	0.0319 (10)	0.0001 (8)	0.0022 (8)	0.0022 (8)
C11	0.0303 (10)	0.0371 (11)	0.0375 (11)	0.0078 (9)	−0.0035 (8)	−0.0029 (9)
C10	0.0305 (10)	0.0305 (11)	0.0400 (11)	0.0062 (8)	−0.0039 (8)	−0.0031 (9)
C4	0.0451 (12)	0.0344 (12)	0.0454 (12)	−0.0009 (10)	−0.0090 (10)	−0.0079 (10)
C8	0.0423 (12)	0.0395 (13)	0.0422 (12)	0.0029 (10)	0.0019 (9)	−0.0033 (10)
C6	0.0362 (11)	0.0361 (12)	0.0506 (13)	−0.0001 (9)	−0.0017 (10)	−0.0040 (10)
C7	0.0401 (12)	0.0386 (13)	0.0520 (14)	−0.0001 (10)	0.0053 (10)	0.0000 (10)
C3	0.0522 (14)	0.0362 (12)	0.0388 (12)	0.0016 (10)	−0.0096 (10)	−0.0056 (10)
O2	0.0313 (7)	0.0307 (8)	0.0478 (9)	0.0042 (6)	0.0070 (6)	0.0025 (7)
N2	0.0277 (8)	0.0356 (9)	0.0334 (9)	−0.0012 (7)	0.0006 (7)	0.0005 (7)
C34	0.0271 (9)	0.0293 (10)	0.0326 (10)	0.0018 (8)	−0.0023 (7)	−0.0032 (8)
C22	0.0288 (9)	0.0308 (11)	0.0314 (10)	−0.0019 (8)	−0.0005 (8)	−0.0003 (8)
C31	0.0316 (10)	0.0304 (10)	0.0299 (10)	−0.0024 (8)	−0.0011 (8)	−0.0017 (8)
C35	0.0263 (9)	0.0323 (11)	0.0340 (10)	−0.0020 (8)	−0.0031 (8)	−0.0005 (8)
C33	0.0269 (9)	0.0310 (10)	0.0308 (9)	−0.0006 (8)	−0.0030 (7)	−0.0015 (8)
C37	0.0297 (9)	0.0321 (10)	0.0355 (10)	0.0041 (8)	−0.0010 (8)	−0.0035 (8)
C38	0.0310 (10)	0.0290 (10)	0.0338 (10)	0.0022 (8)	−0.0038 (8)	0.0009 (8)
C32	0.0295 (9)	0.0313 (10)	0.0335 (10)	−0.0017 (8)	−0.0021 (8)	0.0008 (8)
C29	0.0377 (11)	0.0339 (11)	0.0393 (11)	0.0045 (9)	−0.0039 (9)	−0.0023 (9)
C39	0.0318 (10)	0.0316 (11)	0.0393 (11)	−0.0020 (8)	−0.0014 (8)	0.0025 (9)
C23	0.0330 (10)	0.0346 (11)	0.0371 (11)	0.0023 (8)	−0.0026 (8)	0.0013 (9)
C26	0.0306 (10)	0.0326 (11)	0.0356 (10)	−0.0049 (8)	0.0013 (8)	−0.0033 (8)
C36	0.0273 (9)	0.0369 (11)	0.0327 (10)	−0.0008 (8)	0.0007 (8)	−0.0020 (9)
C25	0.0395 (11)	0.0343 (11)	0.0388 (11)	−0.0047 (9)	0.0056 (9)	0.0040 (9)
C30	0.0339 (10)	0.0302 (11)	0.0357 (10)	−0.0010 (8)	−0.0010 (8)	0.0001 (8)
C24	0.0411 (11)	0.0361 (12)	0.0358 (11)	−0.0005 (9)	−0.0002 (9)	0.0063 (9)
C28	0.0313 (10)	0.0386 (12)	0.0476 (13)	0.0024 (9)	−0.0010 (9)	−0.0102 (10)
C27	0.0325 (10)	0.0385 (12)	0.0420 (12)	−0.0033 (9)	0.0046 (9)	−0.0041 (10)
C20	0.0399 (11)	0.0305 (11)	0.0413 (12)	0.0012 (9)	0.0095 (9)	0.0006 (9)
C19	0.0505 (13)	0.0324 (11)	0.0410 (12)	−0.0076 (10)	0.0032 (10)	−0.0023 (10)
C41	0.0432 (12)	0.0316 (12)	0.0508 (13)	−0.0033 (9)	−0.0066 (10)	0.0017 (10)
C42	0.0459 (12)	0.0361 (12)	0.0460 (13)	−0.0007 (10)	−0.0079 (10)	0.0076 (10)
C40	0.0454 (13)	0.0408 (14)	0.0592 (15)	−0.0052 (10)	0.0102 (11)	0.0109 (12)
C21	0.0492 (13)	0.0378 (12)	0.0306 (10)	0.0013 (10)	0.0008 (9)	0.0008 (9)

### Geometric parameters (Å, °)

**Table:**

O1—C13	1.354 (2)	C31—C26	1.423 (3)
O1—H1A	0.8200	C35—C36	1.394 (3)
N1—C11	1.289 (3)	C35—C39	1.535 (3)
N1—C1	1.419 (3)	C33—C38	1.398 (3)
C14—C15	1.393 (3)	C33—C32	1.450 (3)
C14—C13	1.413 (3)	C37—C38	1.371 (3)
C14—C18	1.535 (3)	C37—C36	1.393 (3)
C13—C12	1.408 (3)	C37—H37	0.9300
C12—C17	1.406 (3)	C38—H38	0.9300
C12—C11	1.452 (3)	C32—H32	0.9300
C9—C8	1.372 (3)	C29—C30	1.371 (3)
C9—C10	1.414 (3)	C29—C28	1.405 (3)
C9—H9	0.9300	C29—H29	0.9300
C15—C16	1.394 (3)	C39—C42	1.532 (3)
C15—H15	0.9300	C39—C40	1.534 (3)
C1—C2	1.373 (3)	C39—C41	1.537 (3)
C1—C10	1.434 (3)	C23—C24	1.410 (3)
C2—C3	1.405 (3)	C23—H23	0.9300
C2—H2	0.9300	C26—C25	1.414 (3)
C16—C17	1.377 (3)	C26—C27	1.421 (3)
C16—H16	0.9300	C36—H36	0.9300
C17—H17	0.9300	C25—C24	1.369 (3)
C5—C4	1.414 (3)	C25—H25	0.9300
C5—C6	1.416 (3)	C30—H30	0.9300
C5—C10	1.424 (3)	C24—H24	0.9300
C18—C19	1.530 (3)	C28—C27	1.364 (3)
C18—C21	1.534 (3)	C28—H28	0.9300
C18—C20	1.545 (3)	C27—H27	0.9300
C11—H11	0.9300	C20—H20A	0.9600
C4—C3	1.364 (3)	C20—H20B	0.9600
C4—H4	0.9300	C20—H20C	0.9600
C8—C7	1.403 (3)	C19—H19A	0.9600
C8—H8	0.9300	C19—H19B	0.9600
C6—C7	1.362 (3)	C19—H19C	0.9600
C6—H6	0.9300	C41—H41A	0.9600
C7—H7	0.9300	C41—H41B	0.9600
C3—H3	0.9300	C41—H41C	0.9600
O2—C34	1.358 (2)	C42—H42A	0.9600
O2—H2A	0.8200	C42—H42B	0.9600
N2—C32	1.290 (3)	C42—H42C	0.9600
N2—C22	1.423 (3)	C40—H40A	0.9600
C34—C33	1.409 (3)	C40—H40B	0.9600
C34—C35	1.414 (3)	C40—H40C	0.9600
C22—C23	1.375 (3)	C21—H21A	0.9600
C22—C31	1.430 (3)	C21—H21B	0.9600
C31—C30	1.416 (3)	C21—H21C	0.9600
			
C13—O1—H1A	109.5	C38—C37—H37	120.2
C11—N1—C1	121.75 (19)	C36—C37—H37	120.2
C15—C14—C13	116.50 (19)	C37—C38—C33	120.41 (19)
C15—C14—C18	122.24 (19)	C37—C38—H38	119.8
C13—C14—C18	121.26 (17)	C33—C38—H38	119.8
O1—C13—C12	119.22 (18)	N2—C32—C33	123.48 (19)
O1—C13—C14	119.22 (17)	N2—C32—H32	118.3
C12—C13—C14	121.56 (18)	C33—C32—H32	118.3
C17—C12—C13	119.24 (19)	C30—C29—C28	120.9 (2)
C17—C12—C11	119.04 (19)	C30—C29—H29	119.6
C13—C12—C11	121.69 (19)	C28—C29—H29	119.6
C8—C9—C10	120.7 (2)	C42—C39—C40	107.66 (19)
C8—C9—H9	119.6	C42—C39—C35	109.20 (17)
C10—C9—H9	119.6	C40—C39—C35	111.78 (18)
C14—C15—C16	123.0 (2)	C42—C39—C41	109.58 (18)
C14—C15—H15	118.5	C40—C39—C41	107.86 (19)
C16—C15—H15	118.5	C35—C39—C41	110.68 (17)
C2—C1—N1	122.5 (2)	C22—C23—C24	121.2 (2)
C2—C1—C10	120.1 (2)	C22—C23—H23	119.4
N1—C1—C10	117.18 (19)	C24—C23—H23	119.4
C1—C2—C3	120.9 (2)	C25—C26—C27	121.36 (19)
C1—C2—H2	119.5	C25—C26—C31	119.75 (19)
C3—C2—H2	119.5	C27—C26—C31	118.9 (2)
C17—C16—C15	119.7 (2)	C37—C36—C35	123.04 (19)
C17—C16—H16	120.1	C37—C36—H36	118.5
C15—C16—H16	120.1	C35—C36—H36	118.5
C16—C17—C12	120.0 (2)	C24—C25—C26	120.6 (2)
C16—C17—H17	120.0	C24—C25—H25	119.7
C12—C17—H17	120.0	C26—C25—H25	119.7
C4—C5—C6	121.6 (2)	C29—C30—C31	120.6 (2)
C4—C5—C10	119.3 (2)	C29—C30—H30	119.7
C6—C5—C10	119.1 (2)	C31—C30—H30	119.7
C19—C18—C21	107.05 (18)	C25—C24—C23	120.1 (2)
C19—C18—C14	111.58 (17)	C25—C24—H24	119.9
C21—C18—C14	110.56 (17)	C23—C24—H24	119.9
C19—C18—C20	107.82 (18)	C27—C28—C29	119.9 (2)
C21—C18—C20	110.30 (17)	C27—C28—H28	120.0
C14—C18—C20	109.47 (17)	C29—C28—H28	120.0
N1—C11—C12	121.45 (19)	C28—C27—C26	121.0 (2)
N1—C11—H11	119.3	C28—C27—H27	119.5
C12—C11—H11	119.3	C26—C27—H27	119.5
C9—C10—C5	118.4 (2)	C18—C20—H20A	109.5
C9—C10—C1	123.20 (19)	C18—C20—H20B	109.5
C5—C10—C1	118.4 (2)	H20A—C20—H20B	109.5
C3—C4—C5	121.0 (2)	C18—C20—H20C	109.5
C3—C4—H4	119.5	H20A—C20—H20C	109.5
C5—C4—H4	119.5	H20B—C20—H20C	109.5
C9—C8—C7	120.8 (2)	C18—C19—H19A	109.5
C9—C8—H8	119.6	C18—C19—H19B	109.5
C7—C8—H8	119.6	H19A—C19—H19B	109.5
C7—C6—C5	121.1 (2)	C18—C19—H19C	109.5
C7—C6—H6	119.4	H19A—C19—H19C	109.5
C5—C6—H6	119.4	H19B—C19—H19C	109.5
C6—C7—C8	119.9 (2)	C39—C41—H41A	109.5
C6—C7—H7	120.0	C39—C41—H41B	109.5
C8—C7—H7	120.0	H41A—C41—H41B	109.5
C4—C3—C2	120.3 (2)	C39—C41—H41C	109.5
C4—C3—H3	119.8	H41A—C41—H41C	109.5
C2—C3—H3	119.8	H41B—C41—H41C	109.5
C34—O2—H2A	109.5	C39—C42—H42A	109.5
C32—N2—C22	118.35 (18)	C39—C42—H42B	109.5
O2—C34—C33	119.24 (18)	H42A—C42—H42B	109.5
O2—C34—C35	119.52 (18)	C39—C42—H42C	109.5
C33—C34—C35	121.23 (18)	H42A—C42—H42C	109.5
C23—C22—N2	122.13 (18)	H42B—C42—H42C	109.5
C23—C22—C31	119.79 (18)	C39—C40—H40A	109.5
N2—C22—C31	118.05 (18)	C39—C40—H40B	109.5
C30—C31—C26	118.67 (19)	H40A—C40—H40B	109.5
C30—C31—C22	122.78 (18)	C39—C40—H40C	109.5
C26—C31—C22	118.55 (19)	H40A—C40—H40C	109.5
C36—C35—C34	116.39 (19)	H40B—C40—H40C	109.5
C36—C35—C39	121.37 (18)	C18—C21—H21A	109.5
C34—C35—C39	122.23 (18)	C18—C21—H21B	109.5
C38—C33—C34	119.41 (18)	H21A—C21—H21B	109.5
C38—C33—C32	118.03 (19)	C18—C21—H21C	109.5
C34—C33—C32	122.54 (18)	H21A—C21—H21C	109.5
C38—C37—C36	119.51 (19)	H21B—C21—H21C	109.5
			
C15—C14—C13—O1	−179.31 (18)	C32—N2—C22—C23	32.8 (3)
C18—C14—C13—O1	0.6 (3)	C32—N2—C22—C31	−149.19 (19)
C15—C14—C13—C12	0.4 (3)	C23—C22—C31—C30	176.7 (2)
C18—C14—C13—C12	−179.71 (18)	N2—C22—C31—C30	−1.4 (3)
O1—C13—C12—C17	179.99 (18)	C23—C22—C31—C26	−3.4 (3)
C14—C13—C12—C17	0.3 (3)	N2—C22—C31—C26	178.49 (17)
O1—C13—C12—C11	−2.0 (3)	O2—C34—C35—C36	−179.94 (18)
C14—C13—C12—C11	178.36 (18)	C33—C34—C35—C36	−1.3 (3)
C13—C14—C15—C16	−0.6 (3)	O2—C34—C35—C39	−1.3 (3)
C18—C14—C15—C16	179.44 (19)	C33—C34—C35—C39	177.37 (18)
C11—N1—C1—C2	26.7 (3)	O2—C34—C33—C38	179.19 (18)
C11—N1—C1—C10	−159.32 (19)	C35—C34—C33—C38	0.5 (3)
N1—C1—C2—C3	174.9 (2)	O2—C34—C33—C32	0.9 (3)
C10—C1—C2—C3	1.1 (3)	C35—C34—C33—C32	−177.72 (18)
C14—C15—C16—C17	0.2 (3)	C36—C37—C38—C33	−1.3 (3)
C15—C16—C17—C12	0.5 (3)	C34—C33—C38—C37	0.8 (3)
C13—C12—C17—C16	−0.8 (3)	C32—C33—C38—C37	179.13 (19)
C11—C12—C17—C16	−178.86 (19)	C22—N2—C32—C33	−178.36 (18)
C15—C14—C18—C19	0.5 (3)	C38—C33—C32—N2	175.45 (19)
C13—C14—C18—C19	−179.45 (18)	C34—C33—C32—N2	−6.3 (3)
C15—C14—C18—C21	119.5 (2)	C36—C35—C39—C42	115.9 (2)
C13—C14—C18—C21	−60.4 (2)	C34—C35—C39—C42	−62.7 (3)
C15—C14—C18—C20	−118.8 (2)	C36—C35—C39—C40	−3.1 (3)
C13—C14—C18—C20	61.3 (2)	C34—C35—C39—C40	178.25 (19)
C1—N1—C11—C12	−171.02 (18)	C36—C35—C39—C41	−123.4 (2)
C17—C12—C11—N1	177.58 (19)	C34—C35—C39—C41	58.0 (2)
C13—C12—C11—N1	−0.5 (3)	N2—C22—C23—C24	−179.59 (19)
C8—C9—C10—C5	0.5 (3)	C31—C22—C23—C24	2.4 (3)
C8—C9—C10—C1	−178.3 (2)	C30—C31—C26—C25	−177.89 (19)
C4—C5—C10—C9	−179.6 (2)	C22—C31—C26—C25	2.2 (3)
C6—C5—C10—C9	−0.2 (3)	C30—C31—C26—C27	2.2 (3)
C4—C5—C10—C1	−0.7 (3)	C22—C31—C26—C27	−177.73 (19)
C6—C5—C10—C1	178.65 (19)	C38—C37—C36—C35	0.5 (3)
C2—C1—C10—C9	178.7 (2)	C34—C35—C36—C37	0.8 (3)
N1—C1—C10—C9	4.6 (3)	C39—C35—C36—C37	−177.88 (19)
C2—C1—C10—C5	−0.1 (3)	C27—C26—C25—C24	180.0 (2)
N1—C1—C10—C5	−174.25 (18)	C31—C26—C25—C24	0.1 (3)
C6—C5—C4—C3	−178.7 (2)	C28—C29—C30—C31	−0.3 (3)
C10—C5—C4—C3	0.6 (3)	C26—C31—C30—C29	−1.4 (3)
C10—C9—C8—C7	−0.4 (3)	C22—C31—C30—C29	178.5 (2)
C4—C5—C6—C7	179.1 (2)	C26—C25—C24—C23	−1.2 (3)
C10—C5—C6—C7	−0.3 (3)	C22—C23—C24—C25	−0.1 (3)
C5—C6—C7—C8	0.4 (4)	C30—C29—C28—C27	1.3 (3)
C9—C8—C7—C6	−0.1 (4)	C29—C28—C27—C26	−0.5 (3)
C5—C4—C3—C2	0.3 (4)	C25—C26—C27—C28	178.8 (2)
C1—C2—C3—C4	−1.2 (4)	C31—C26—C27—C28	−1.2 (3)

### Hydrogen-bond geometry (Å, °)

**Table:**

*D*—H···*A*	*D*—H	H···*A*	*D*···*A*	*D*—H···*A*
O1—H1A···N1	0.82	1.81	2.552 (2)	150.
O2—H2A···N2	0.82	1.89	2.631 (2)	150.
C20—H20B···O1	0.96	2.38	2.998 (3)	121.
C21—H21B···O1	0.96	2.35	2.987 (3)	124.
C41—H41C···O2	0.96	2.33	2.979 (3)	124.
C42—H42B···O2	0.96	2.41	3.056 (3)	124.
